# A photocleavable peptidic Ru(ii) mass-tag enabling targeted DESI and MALDI mass spectrometry imaging in cancer tissues

**DOI:** 10.1039/d6sc00953k

**Published:** 2026-04-13

**Authors:** Mihyun Park, Juliana P. L. Gonçalves, Sandra Deiser, Sebastian Fenzl, Felix A. Böhm, Katja Steiger, Susanne Kossatz, Nicole Strittmatter, Angela Casini

**Affiliations:** a Chair of Medicinal and Bioinorganic Chemistry, Department of Chemistry, School of Natural Sciences, Technical University of Munich 85748 Garching Germany angela.casini@tum.de; b Professorship of Analytical Chemistry, Department of Bioscience, School of Natural Sciences, Technical University of Munich 85748 Garching Germany; c Institute of Pathology, School of Medicine and Health, Technical University of Munich 81675 Munich Germany; d Chair of Pharmaceutical Radiochemistry, Department of Chemistry, TUM School of Natural Sciences, Technical University of Munich 85748 Garching Germany; e Comparative Experimental Pathology, Institute of Pathology, School of Medicine and Health, Technical University of Munich 81675 Munich Germany; f Department of Nuclear Medicine, TUM University Hospital, and Central Institute for Translational Cancer Research (TranslaTUM), School of Medicine and Health, Technical University of Munich 81675 Munich Germany

## Abstract

Mass spectrometry imaging (MSI) is a versatile technique for spatially resolved multi-omics analysis in clinical research. However, its proteomic applications remain constrained by issues such as poor ionisation efficiency and signal interference from complex tissue matrices. On-tissue mass-tag labelling (OTMT) overcomes these challenges by employing affinity-based imaging agents that carry cleavable, highly ionisable reporter groups known as mass-tags (MTs). In this work, we introduce TATE-Ru(ii)MT, which comprises a somatostatin receptor subtype 2 (SSTR2)-targeting cyclic peptide and a photocleavable Ru(ii) polypyridine complex. We demonstrate that this MT enables multimodal visualisation of SSTR2 in tissue using matrix-assisted laser desorption/ionisation (MALDI) and desorption electrospray ionisation (DESI) MSI, following a staining workflow fully compatible with the widely used, commercially available Miralys™ MTs from AmberGen. Unlike commercial MTs, which use peptide sequences as photocleavable reporter groups, Ru(ii)-based MTs are not restricted to MALDI-MSI, offering a flexible platform with variable MT design and broad methodological applicability.

## Abstract 2

Mass-tag with photocleavable Ru(ii) complex and peptidic binding motif enables bimodal SSTR2 imaging *via* MALDI- and DESI-MSI, compatible with commercial antibody-based mass-tags for multiplex imaging of disease-relevant proteins in tissue.

## Graphical abstract

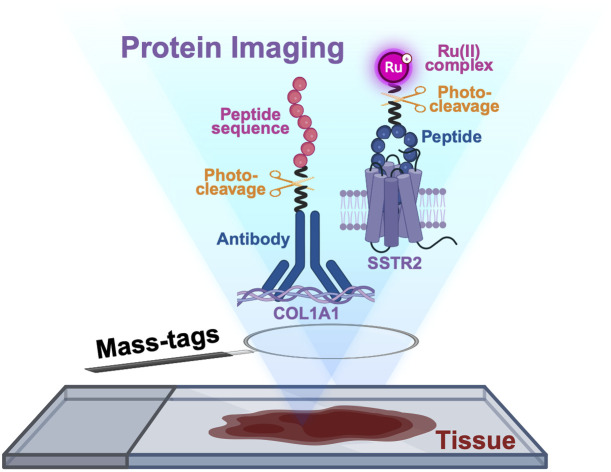

## Introduction

Since the concept of “personalised medicine” was introduced in 1999,^1^ cancer research and diagnostics have undergone a profound paradigm shift away from the traditional “one-size-fits-all” approach and toward strategies aimed at improving early detection and patient-centric treatment. In 2015, the launch of the “Precision Medicine Initiative” marked a pivotal moment, with the terminology changing from “personalised medicine” to “precision medicine”.^2^ Precision medicine considers variability in genetic, socio-environmental and lifestyle factors within subpopulations to design targeted therapies.^3^ Achieving this goal requires the collection and analysis of large-scale patient data, supported by comprehensive multi-omics approaches and the identification of biomarkers as an avenue to decide on the appropriate personalised therapy.

One promising strategy is mass spectrometry imaging (MSI), a surface-imaging technique typically performed *ex vivo* that enables simultaneous visualisation of the spatial distribution of a broad range of endogenous and exogenous molecules across diverse biological samples.^4–6^ Unlike traditional bulk omics methods, which lose cellular context and heterogeneity, MSI provides two- and three-dimensional spatial omics data,^7^ offering critical insights into the complex mechanisms underlying cellular function, disease progression, and therapeutic response.^8–13^ In MSI, the sample surface is scanned along a predefined raster pattern using a desorption–ionisation source, generating a mass spectrum at each defined pixel. The spatial distribution of each detected molecular species can then be visualised in ion density maps. Depending on the ionisation source and the instrumental configuration, MSI often requires minimal to no sample preparation and can achieve various spatial and depth resolutions without sample destruction. Due to its label-free nature, MSI has gained popularity as a powerful tool for spatial metabolomics and lipidomics.^14–16^ The minimally destructive nature allows subsequent histological assessments on the same sample, which is crucial for precious clinical samples.^17^ In clinical research application, the two most commonly applied ionisation sources are matrix-assisted laser desorption/ionisation (MALDI) and desorption electrospray ionisation (DESI).^6,18^

However, when it comes to low-abundant proteins, MSI still struggles to identify them. The high molecular weight and overall complexity of proteins lead to low ionisation efficiency, poor desorption, and ion suppression by more abundant endogenous components, resulting in insufficient sensitivity.

One strategy for high-sensitivity and high-specificity intact protein detection is on-tissue mass-tag labelling (OTMT).^22^ Mass-tags (MTs) are affinity-based imaging agents generally composed of a target-specific binding motif linked to a cleavable, highly ionisable reporter group. Commonly, photocleavable organic molecules act as reporter groups, while antibodies (Abs) serve as binding motifs, leveraging well-established methodologies derived from immunohistochemistry (IHC). Compared to conventional histological methods, OTMT offers near-unlimited multiplexing potential, as any cleavable and ionisable molecule combined with a target-specific binding motif can serve as a MT. Furthermore, through optimised washing and sample-handling protocols, OTMT can preserve MSI's multi-omics and multimodal capabilities.^21,23^

**Fig. 1:**
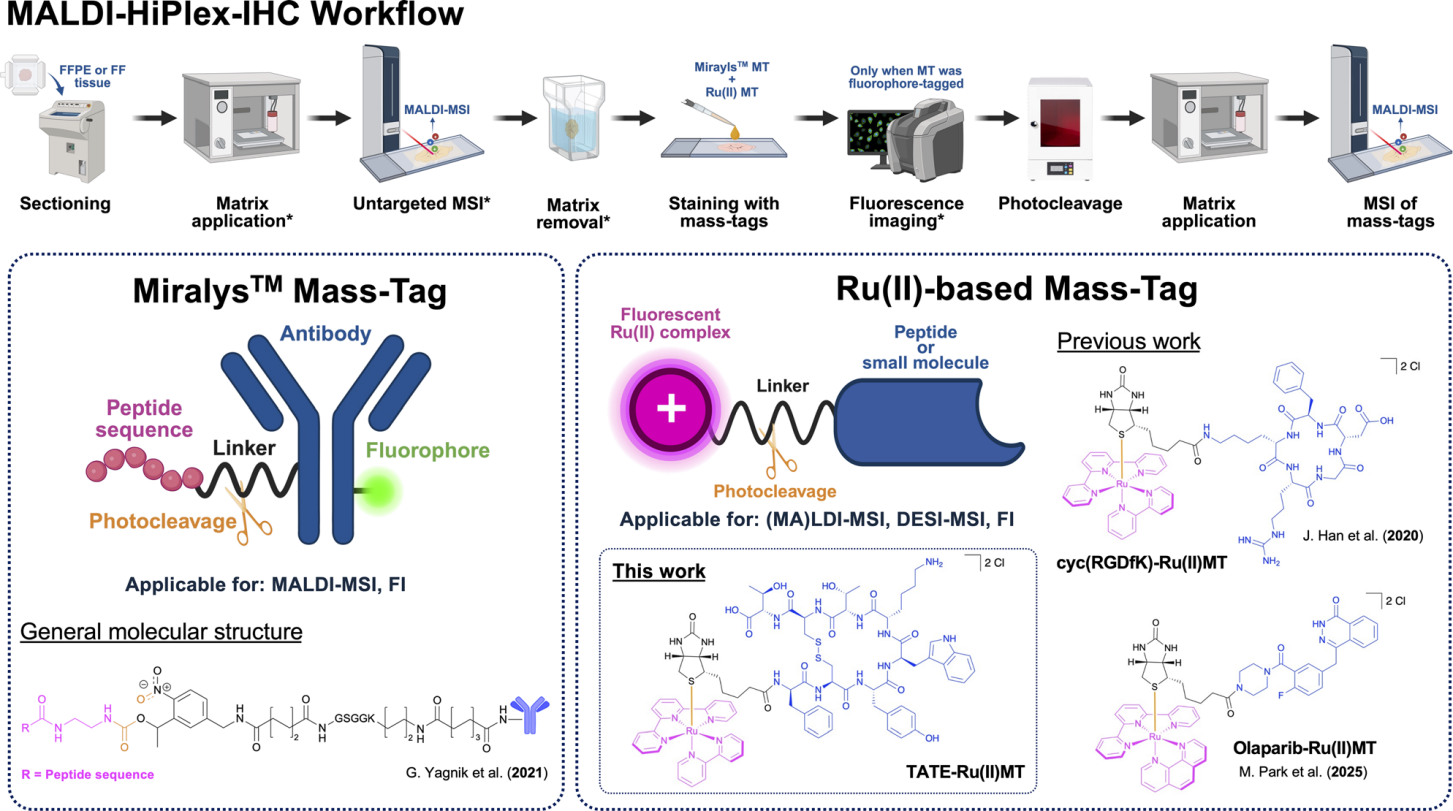
General overview of the MALDI-HiPlex-IHC workflow and structural comparison of Miralys™ MTs with Ru(ii)-based MTs. The general MALDI-HiPlex-IHC workflow is illustrated with optional steps marked by an asterisk. The optional steps for the untargeted acquisition and fluorescence imaging are used only when relevant, and their specific sequence depends on the sample state (FFPE *vs.* FF) and the analytes of interest.^19^ In the clinically used protocol, the staining of the tissue is conducted with the Miralys™ MTs, while in our adapted protocol, we added both a Miralys™ and a Ru(ii) MT. In the structural comparison, reporter groups are indicated in pink, linkers in black, binding motifs in blue, and photocleavage sites in orange.^19–21^ Abbreviations: FFPE = formalin-fixed paraffin-embedded, FF = fresh-frozen, FI = fluorescence imaging. The figure was partially created using BioRender (https://BioRender.com/6q7jwpc, https://BioRender.com/o54vaod, and https://BioRender.com/w1j0pf3).

A prominent example is the commercially available Miralys™ MTs by AmberGen Inc. (Billerica, MA, USA) for MALDI-MSI applications,^24^ which consist of Abs modified with photocleavable peptide sequences as reporter groups (Fig. 1).^19,25^ Since their introduction, these MTs have been increasingly applied in clinical research for targeted protein expression profiling in tissue.^23,26^ This technology has recently been implemented in the sequential acquisition of eight MSI analyte modalities (metabolites in positive and negative mode, lipids in positive and negative mode, *N*-linked glycans, *O*-linked *N*-acetylglucosamine, small intact proteins, and tryptic peptides) from the same tissue section, providing a broad range of spatial molecular information using a single instrument.^27^

Although, these highly-selective Ab-based MTs with peptide reporter groups have proven effective for visualising clinically relevant proteins in tissue samples, their use can still present several challenges: (1) the relatively large size of the photocleavable peptide sequences (>1000 Da) restricts their application primarily to MALDI-MSI and no successful report of their use with other ionisation platforms have been made to date; (2) unambiguous identification of the organic reporter groups can be challenging, *e.g.*, due to isobaric overlaps with endogenous molecules, necessitating extensive pre-washing steps or fragmentation analysis;^25,28^ (3) the use of Abs often results in high costs, stringent handling requirements, and limited storage stability.

In 2020, our group reported the first metal-based MT, cyc(RGDfK)-Ru(ii)MT, consisting of a cyclic peptide as the integrin-targeting binding motif and a photocleavable Ru(ii) polypyridine complex as the reporter group (Fig. 1).^20^ In this proof-of-concept study, we demonstrated that Ru(ii)MT can visualise the spatial distribution of ανβ3 integrin in fresh-frozen hypopharyngeal tumour sections from a head and neck cancer patient using laser desorption ionisation (LDI) MSI. More recently, we developed a Ru(ii)MT, Olaparib-Ru(ii)MT, featuring a small-molecule inhibitor as a PARP1-binding motif for MSI (Fig. 1).^21^ In this study, we demonstrated that Ru-based MTs can be used for DESI-MSI, extending the MT concept to ambient MSI techniques. Furthermore, by leveraging the intrinsic luminescent properties of Ru(ii) polypyridine complexes, we showed that these MTs can also be utilised for fluorescence imaging, enabling a bimodal readout from the same sample.^21^

The incorporation of photocleavable Ru(ii) polypyridine complexes as reporter groups provides key advantages over other MT families.^22^ For example, the natural isotope pattern of Ru enables unambiguous identification of reporter group signals in complex datasets through distinctive isotope patterns without requiring fragmentation. The ligand structure of Ru(ii) polypyridine complexes is highly tunable, granting access to an extensive library (hundreds of compounds with different chelating N^N ligands and substituents) of Ru-based reporter groups for multiplexing.^29^ Moreover, due to their small size and intrinsic charge after photocleavage, Ru(ii) reporter groups exhibit good desorption and high ionisation efficiency on tissues.

**Scheme 1:**
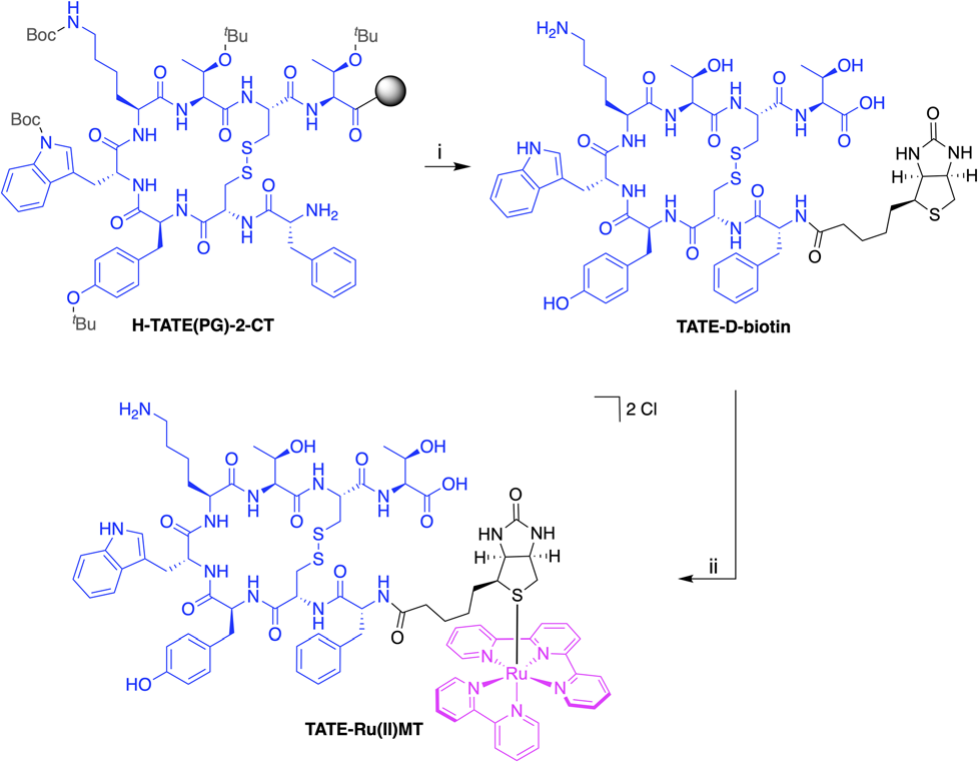
Synthesis of TATE-Ru(ii)MT. Reagents and reaction conditions: (i) (1) addition of d-biotin with HATU/HOAt/DIPEA in DMF at RT for 30 min; (2) cleavage from the resin and global deprotection with TFA/TIPS/H_2_O at RT for 45 min and with TFA at RT o.n.; 26% yield (ii) ligand exchange reaction with [RuCl(bpy)(tpy)]PF_6_ in H_2_O at RT under inert atmosphere o.n.; 34% yield.

Here, we broadened the scope of the Ru(ii)-based MTs, designing a novel TATE-Ru(ii)MT (Scheme 1), featuring a cyclic peptide targeting the somatostatin receptor subtype 2 (SSTR2), a membrane protein frequently overexpressed in neuroendocrine tumours and extensively studied as a diagnostic and therapeutic target.^30–34^ Specifically, we conjugated the peptide Tyr^3^-octreotate (TATE) to a d-biotin moiety, which further coordinated to a [Ru(bpy)(tpy)]^2+^ (bpy = 2,2′-bipyridine, tpy = 2,2′:6′,2″-terpyridine) fragment (Scheme 1). We demonstrate that TATE-Ru(ii)MT can be integrated into the MALDI-HiPlex-IHC workflow,^19,25^ enabling visualisation of SSTR2, currently not part of the Miralys™ portfolio, alongside the commercially available Ab-based MTs using MALDI-MSI.

Additionally, TATE-Ru(ii)MT can be used with DESI-MSI, which offers several advantages as an ambient ionisation technique. For example, it does not require high vacuum or matrix application, reducing the number of sample preparation steps and the risk of artefact introduction. Previously, only one other MT design utilising pH-sensitive cleavable linkers has been demonstrated for detection with DESI-MSI,^35^ underscoring the need for further MT development to expand the current toolbox of multiplexed tissue imaging.

## Results and discussions

TATE-Ru(ii)MT was synthesised *via* a two-step procedure (Scheme 1). In the first step, d-biotin was conjugated to the *N*-terminus of TATE through an amidation reaction. The successful synthesis was confirmed by analytical high-performance liquid chromatography (HPLC, Fig. S1) and high-resolution mass spectrometry (HRMS, Fig. S2). In the second step, the resulting biotinylated conjugate was coupled to the Ru(ii) reporter group *via* a ligand exchange reaction, wherein the chloride ligand of [RuCl(bpy)(tpy)]Cl was replaced by the thioether moiety of d-biotin (detailed experimental procedures are provided in the SI). A purity of ≥95% was verified for TATE-Ru(ii)MT by analytical HPLC (Fig. S3) and HRMS, identifying [M]^2+^ as the predominant species (Fig. S4).

The affinity of TATE-Ru(ii)MT for SSTR2 was evaluated through *in vitro* competitive binding assays using CHO/sst2 cells. [^125^I]I-TOC served as the radiolabelled competitor (detailed experimental procedures are provided in the SI). TATE-Ru(ii)MT exhibited a low nanomolar binding affinity with a half-maximal inhibitory concentration (IC_50_) of 2.50 ± 0.22 nM, matching that of the standard Lu-DOTA-TATE (IC_50_ = 11.3 ± 1.5 nM; Fig. S5 and S6).

Afterwards, we evaluated the ability of TATE-Ru(ii)MT to visualise SSTR2 in fresh-frozen tissues using MALDI-MSI and DESI-MSI. SSTRs are expressed in various neoplasms,^30^ and among the six subtypes, SSTR2 is the most frequently expressed in lung carcinoids.^36^ Beyond cancer cells, somatostatin is primarily produced in the central nervous system, gastrointestinal tract, and endocrine glands, where it exerts inhibitory effects on hormone release. Somatostatin also plays a role in immune regulation,^37–39^ and SSTRs are highly expressed in monocytes, monocyte-derived macrophages, and dendritic cells.^40^

For this study, a xenograft tumour derived from the human small cell lung carcinoma (SCLC) cell line NCI-H446 was selected as a positive control due to its high SSTR2 expression. Murine spleen samples served as negative controls, as previous studies indicate that healthy murine spleen exhibits low or no SSTR2 expression.^41–43^ To evaluate the applicability of TATE-Ru(ii)MT, the samples were stained under comparable conditions for both MALDI- and DESI-MSI, following the MALDI-HiPlex-IHC workflow (experimental details provided in the SI). For MALDI-MSI, sections were stained with a solution containing TATE-Ru(ii)MT and the Ab-based Miralys™ MT targeting an architectural marker for collagen COL1A1 to verify that TATE-Ru(ii)MT can be integrated into the multiplex staining workflow of MALDI-HiPlex-IHC.^19,25^ Since Ab-based MTs require precise dilution and incubation times for reliable staining, the concentration (10 µg mL^−1^) and staining duration (overnight at 4 °C in a humidified chamber) of TATE-Ru(ii)MT were aligned with those of COL1A1-MT (12 µg mL^−1^) (detailed experimental procedures are provided in the SI). After staining, the reporter groups were photocleaved using the AmberGen Light Box.

For DESI-MSI, a higher concentration of TATE-Ru(ii)MT (100 µg mL^−1^) was used. Due to the higher concentration, tissue sections were stained for only 1 h under conditions identical to those used for MALDI-MSI. As a negative control, a longitudinal section of murine spleen was used. Photocleavage conditions were adjusted according to a previously established protocol for DESI-MSI,^21^ ensuring efficient cleavage of the Ru(ii) reporter group.

**Fig. 2:**
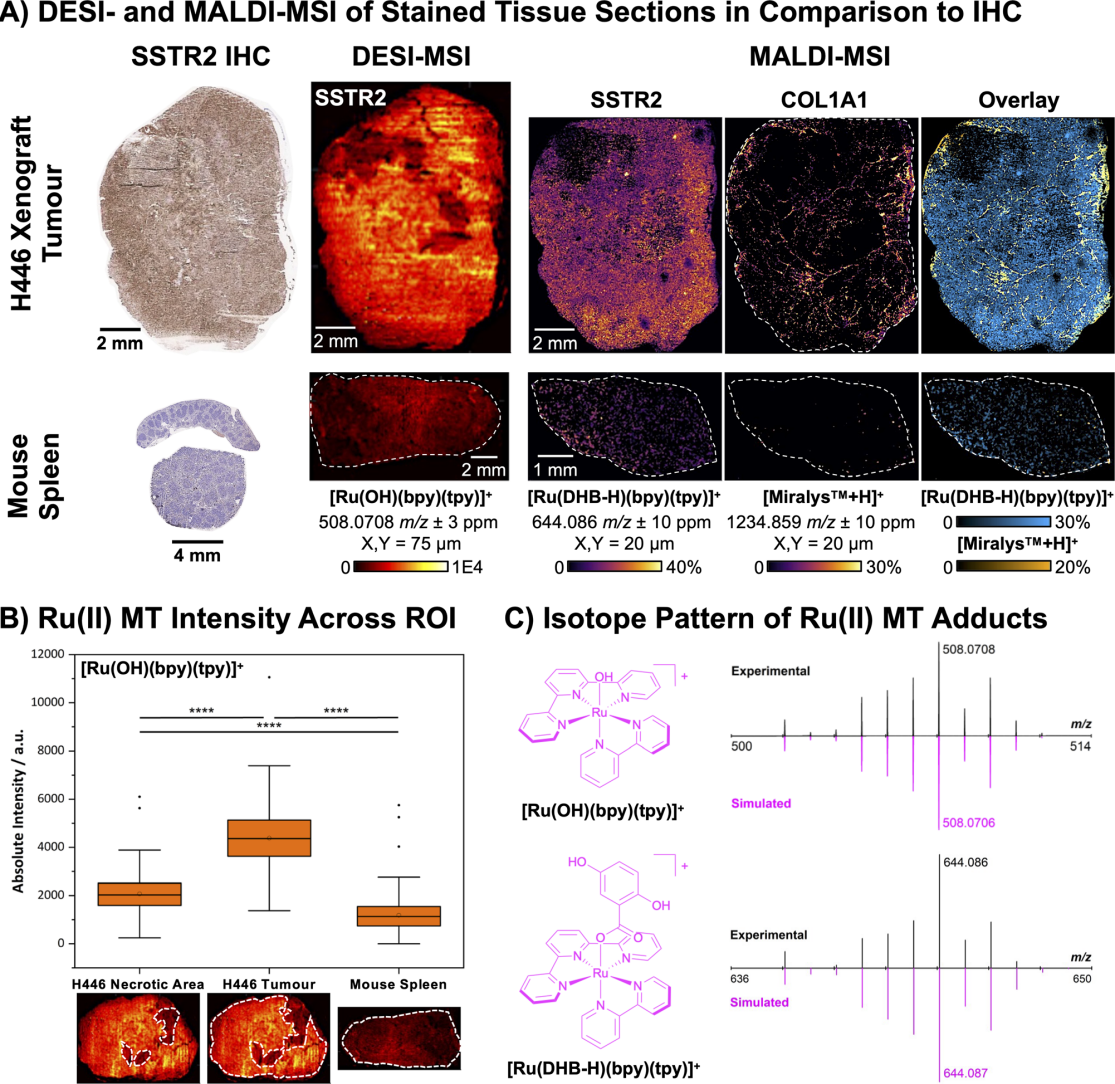
(A) DESI- and MALDI-MSI of stained tissue sections compared to SSTR2 IHC. Samples include H446 xenograft tumour (SSTR2-positive control) and murine spleen (SSTR2-negative control). DESI-MSI (*X*, *Y* = 75 µm, total ion current (TIC) normalised): H446 xenograft tumour and longitudinal murine spleen sections were stained with 100 µg mL^−1^TATE-Ru(ii)MT, following the MALDI-HiPlex-IHC workflow. The main reporter group adduct observed were [Ru(OH)(bpy)(tpy)]^+^ (508.0708 ± 3 ppm) and [Ru(N_2_)(bpy)(tpy)]^2+^ (Fig. S6). MALDI-MSI (*X*, *Y* = 20 µm, TIC-normalised and low-denoised): H446 xenograft tumour section and murine spleen cross-section were stained with 10 µg mL^−1^TATE-Ru(ii)MT and 12 µg mL^−1^ COL1A1-MT, following the MALDI-HiPlex-IHC workflow. Main reporter group adducts observed were [Ru(DHB-H)(bpy)(tpy)]^+^ (644.086 ± 10 ppm) for TATE-Ru(ii)MT and [Miralys™ + H]^+^ (1234.859 ± 10 ppm) and [Miralys™ + Na]^+^ (Fig. S7) for COL1A1-MT. Overlay image of both reporter group adducts shows the spatial distribution of both targets in a single image (blue: [Ru(DHB-H)(bpy)(tpy)]^+^; orange: [Miralys™ + H]^+^). In both modalities, SSTR2 expression was high in intact tumour regions, lower in necrotic areas of the tumour, and minimal in spleen samples, consistent with IHC results. (B) Ru(ii) MT intensity across ROI. To assess selective staining by TATE-Ru(ii)MT, necrotic tumour, intact tumour, and spleen regions were marked as region of interests (ROI), and the absolute intensity of [Ru(OH)(bpy)(tpy)]^+^ (508.0708 ± 3 ppm) in each pixel (*X*, *Y* = 75 µm) in each ROI were plotted as box plots. Two-sample *t*-tests indicated significant differences between the selected ROI (*p* < 0.0001), confirming the target selectivity of TATE-Ru(ii)MT. (C) Isotope pattern of Ru(ii) MT adducts. The chemical structure and the experimental and simulated isotope patterns of the Ru(ii) reporter group adducts are depicted.

In both MSI methodologies, a high abundance of the Ru(ii) reporter group was observed in intact tumour regions, while significantly lower abundance was detected in the necrotic areas of the tumour and the spleen samples (Fig. 2A and B), consistent with the IHC results (Fig. 2A, S7 and S8). These findings confirm that TATE-Ru(ii)MT is compatible with both MALDI- and DESI-MSI, and that the clinically and commercially established MALDI-HiPlex-IHC workflow can be successfully adapted for TATE-Ru(ii)MT. In MALDI-MSI, the predominant adduct of the Ru(ii) reporter group was [Ru(DHB-H)(bpy)(tpy)]^+^, resulting from incorporation of a DHB matrix molecule (Fig. 2A). In DESI-MSI, the main adducts observed were [Ru(N_2_)(bpy)(tpy)]^2+^ and [Ru(OH)(bpy)(tpy)]^+^, showing same spatial distribution (Fig. 2A and S9). Overlay images of the reporter group signals from COL1A1-MT, detected as the [M + H]^+^ and [M + Na]^+^ adducts (Fig. S10), and TATE-Ru(ii)MT clearly demonstrate that bimodal staining using Ab-based and peptide-based MTs is feasible, highlighting the potential for integrating TATE-Ru(ii)MT into the multiplex AmberGen panel (Fig. 2A). Although a clear distinction is observed between positive and negative controls, low-level non-specific staining persists in the negative controls (Fig. 2B). Notably, comparable non-specific staining in the red pulp can also be observed in SSTR2 IHC of murine and rat spleen (Fig. S7 and S8). Potential strategies for improvement include reducing staining concentration, incorporating additional washing steps, or refining peptide structural design to enhance target selectivity.

## Conclusions

In this study, we introduce TATE-Ru(ii)MT targeting the SSTR2 for MSI. We demonstrate that Ru(ii)-based MTs can be integrated into the multiplex MALDI-HiPlex-IHC workflow offered by Bruker and AmberGen, enabling their incorporation into routine MSI workflows for spatial proteomic analysis in clinical research. Compared to commercially available Miralys™ MTs, Ru(ii)-based MTs offer several advantages, making them a valuable addition to the existing portfolio. As the choice of binding motif is not restricted to Abs, this approach provides great flexibility in MT design tailored to specific targets of interest, and peptides also feature a higher tissue penetration.

Peptide-based targeting offers a high degree of versatility, although it is not as “universally” applicable as Ab-based approaches. While Abs can, in principle, be generated against nearly any antigen, peptides generally rely on the presence of specific binding pockets or receptor sites that have evolved to interact with endogenous ligands. Presently, hundreds of validated and well-documented peptide sequences are available for MT development (*e.g.*, ∼170 peptides in active clinical development, with many more in preclinical stages).^44^ Importantly, the applicability of peptides is rapidly expanding through advanced discovery strategies such as phage display and computational bioinformatics, which enable high-throughput screening of billions of random peptide sequences against a target.^44^

In addition, Ru(ii) MTs are synthesised *via* well-established procedures, enabling cost-effective, straightforward manufacturing, and can be stored as solids, reducing time-dependent degradation and increasing shelf life. Moreover, since small Ru(ii) complexes with high ionisation efficiency and unique isotopic pattern distribution are applied as reporter groups, their application extends beyond LDI-MSI,^20^ and DESI-MSI,^21^ to MALDI-MSI, broadening the accessibility of the MT concept to the MSI community.

## Author contributions

M. P. synthesized and analyzed TATE-d-biotin and TATE-Ru(ii)MT, designed and conducted the MSI experiments, analyzed the data, and wrote the manuscript. M. P. and J. P. L. G. conducted the MALDI-MSI experiments. S. D. synthesized TATE and Lu-DOTA-TATE. S. F. and M. P. performed the SSTR2 IC_50_ assay. F. A. B. assisted in the HPLC purification of TATE-Ru(ii)MT. K. S. performed the IHC staining and slide scanning. S. K. provided advice on the selection of tissue samples and provided them. N. S. and A. C. conceived the research, supervised the project, secured funding, and provided essential resources. All authors contributed to the manuscript through critical review and approved the final version of the manuscript.

## Conflicts of interest

There are no conflicts to declare.

## Supplementary Material

SC-OLF-D6SC00953K-s001

## Data Availability

The data supporting this article have been included as part of the supplementary information (SI). In addition, MSI data files discussed in this work have been provided on Zenodo: https://doi.org/10.5281/zenodo.18419727. Supplementary information: experimental protocols and SI figures. See DOI: https://doi.org/10.1039/d6sc00953k.
