# Supplementary material for: A photocleavable peptidic Ru(ii) mass-tag enabling targeted DESI and MALDI mass spectrometry imaging in cancer tissues
Source: Chem Sci. 2026 Apr 13;17(21):10504–10. doi: 10.1039/d6sc00953k (PMC13088906; doi:10.1039/d6sc00953k)
Supplement: SC-017-D6SC00953K-s001 [file SC-017-D6SC00953K-s001.pdf]

Supplementary Information (SI) for Chemical Science.
This journal is © The Royal Society of Chemistry 2026

Supporting Information

A photocleavable peptidic Ru(II) mass-tag enabling targeted DESI and MALDI mass spectrometry imaging in cancer tissues

Materials and Methods

Synthesis Protocols

General solvents and reagents were purchased from commercial suppliers and used without further purification unless otherwise stated. H-TATE(PG)-2-CT and [RuCl(bpy)(tpy)]PF_6 were prepared as previously reported.[1, 2]

TATE-D-biotin

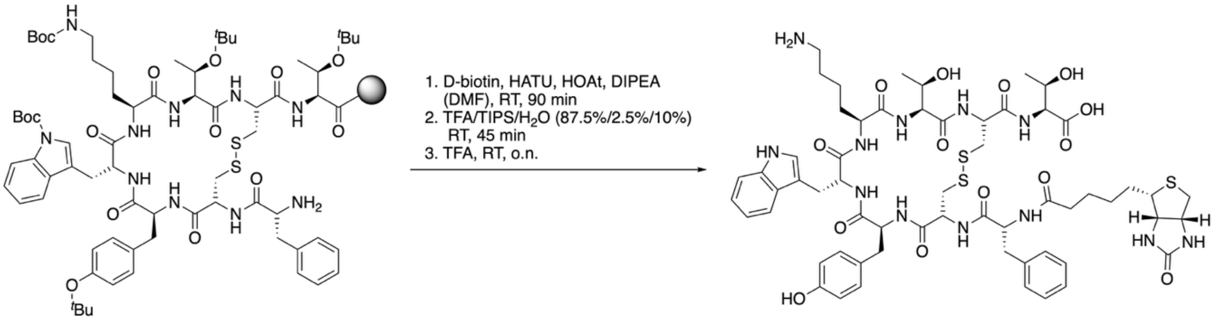

The resin-bound cyclic peptide, H-TATE(PG)-2-CT (40 µmol, 1.0 equiv.), was agitated in NMP (5 mL) for 30 min at RT and washed with DMF (6 x 5 mL). D-biotin (60 µmol, 1.5 equiv.), HATU (60 µmol, 1.5 equiv.), and HOAt (60 µmol, 1.5 eq.) were dissolved in DMF (3 mL) and pre-activated with DIPEA (80 µmol, 2.0 equiv.) for 15 min in an ultrasonic bath. The resulting reaction mixture was added to the resin and agitated for 90 min. Afterwards, the peptide was washed with DMF (6 x 5 mL) and DCM (4 x 5 mL). For the cleavage of peptide from the resin and deprotection of acid-labile protection groups, a cleavage cocktail of TFA/TIPS/H_2O (87.5%/2.5%/10%, 5 mL) was added to the resin and agitated for 45 min at RT. The solution with the deprotected TATE-biotin was collected in a round-bottom flask, and the remaining resin was washed with TFA (5 mL). The TFA washing solution was combined with the deprotected TATE-biotin and stirred at RT overnight. The solution was concentrated under a N_2 stream. The crude product was diluted in MeCN/H_2O (1:1, 500 µL) and purified via semi-preparative RP-HPLC. After purification via RP-HPLC, the solvent was removed under reduced pressure. The residue was redissolved in ^{t}BuOH/H_2O (*v/v* = 1:1), frozen at -80 °C and lyophilised, affording TATE-D-biotin in 26% yield (11 µmol).

RP-HPLC (20-50% eluent B, 20 min, λ = 220 nm): t_R = 17.2 min.
DESI-HRMS: Calcd. for $C_{59}H_{78}N_{12}O_{14}S_3$ $[M+2H]^{2+}$: *m/z* = 638.2534; Found: *m/z* = 638.2531.

TATE-Ru(II)MT – [Ru(TATE-biotin)(bpy)(tpy)]Cl_2

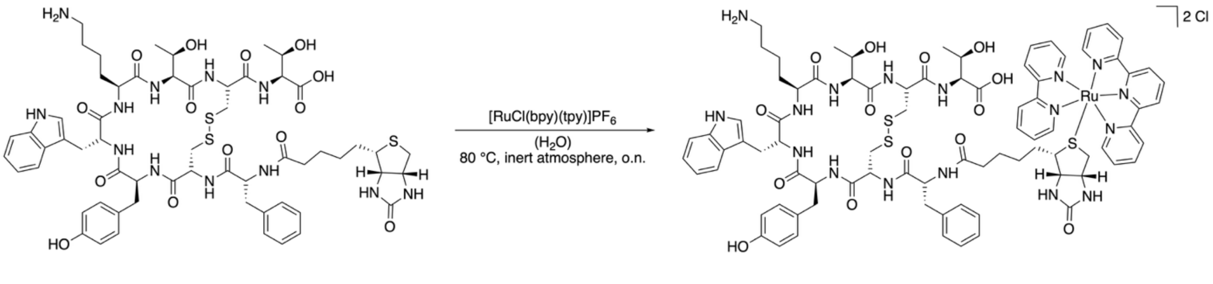

TATE-biotin (13 mg, 10 µmol, 1.0 equiv.) and [RuCl(bpy)(tpy)]PF_6 (7.8 mg, 12 µmol, 1.1 equiv.) were dissolved in H_2O (2 mL) and stirred overnight at 80 °C in the dark. The solvent was removed under reduced pressure. The crude product was purified via semi-preparative RP-HPLC. After purification via RP-HPLC, the solvent was removed under reduced pressure. The residue was redissolved in ^{t}BuOH/H_2O (*v/v* = 1:1), frozen at -80 °C and lyophilised, affording TATE-Ru(II)MT in 34% yield (6.6 mg, 3.59 µmol).

RP-HPLC (30-60% eluent B, 20 min, λ =220 nm): t_R = 8.2 min.
DESI-HRMS: Calcd. for $C_{84}H_{97}N_{17}O_{14}RuS_3$ $[M]^{2+}$: *m/z* = 882.7798; Found: *m/z* = 882.7788.

Reversed-Phase High-Performance Liquid Chromatography

Analytical and semi-preparative RP-HPLC were carried out on Shimadzu Corp. Instruments equipped with two LC-20AD gradient pumps (Shimadzu Scientific Instruments, Kyoto, Japan), a CBM-20A communications module (Shimadzu Scientific Instruments, Kyoto, Japan) and a Smartline UV detector 2500 (λ = 220 nm, 254 nm; Knauer Wissenschaftliche Geräte GmbH, Berlin, Germany). A MultoKrom 100–5 C18 (125 x 4.6 mm, 5 µm particle size; CS-Chromatographie Service GmbH, Langerwehe, Germany) with a flow rate of 1.0 mL/min was used for analytical RP-HPLC and a MultoKrom 100–5 C18 (250 x 20.0 mm, 5 µm particle size; CS-Chromatographie Service GmbH, Langerwehe, Germany) with a flow rate of 10 mL/min was used for semi-preparative RP-HPLC. Different gradients of A (H_2O + 0.1% TFA) and B (MeCN + 5% H_2O and 0.1% TFA) were used as eluents for all RP-HPLC operations. All compounds are >95% pure by HPLC analysis.

High-Resolution Mass Spectrometry

High-resolution mass spectra (HRMS) were recorded on a Q Exactive™ Plus Hybrid Quadrupole-Orbitrap™ mass spectrometer (Thermo Fisher Scientific, Bremen, Germany) using a desorption electrospray ionisation (DESI) source and following parameters:

- Mass spectrometer: 70,000 resolution at *m/z* 200, positive ion mode, 1 microscan, no lock mass, 5E6 AGC target, 250 ms maximum injection time, 320 °C capillary temperature, 100 S-lens RF level
- DESI source: Sprayer-to-sample distance of 1.5 mm, sprayer-to-inlet distance of 6 mm, spray angle at 75°, and collection angle at 10°, 4.5 kV spray voltage, 1.5 µL/min spray flow rate, 7 bar nebulising gas pressure (N_2 purity N5.0), 95% MeOH as spray solvent.

Thermo Xcalibur 3.0.63 (Thermo Fischer Scientific, Waltham, USA) and OriginPro 2021 9.8.0.200 (Origin Lab Corp., Northampton, USA) were used for data processing. Theoretical isotope fine structures were calculated with enviPat Web,[3] and created with OriginPro 2021 9.8.0.200 (Origin Lab Corp., Northampton, USA).

SSTR2 IC_{50} Assay

In vitro competitive studies were performed on CHO/sst2 cells (provided by Dr. Jenny Koenig, University of Cambridge, Cambridge, UK) using [^{125}I]I-TOC as the competitor and Lu-DOTA-TATE (provided by Dr. Sandra Deiser, TUM, Munich, Germany) as the clinical standard. For the assay, solutions of Lu-DOTA-TATE and TATE-Ru(II)MT in HBSA (Hanks balanced salt solution + 1% bovine serum albumin) were prepared at concentrations ranging from 1 x 10^{-10} to 1 x 10^{-4} M.

CHO/sst2 were seeded in 24-well plates (1 x 10^5 cells/well) and incubated in 1 mL DMEM/F12 GlutaMax plus 10% FCS medium at 37 °C 24 ± 2 h before the experiment. On the day of the experiment, the culture medium was removed, and each cell lawn was washed with 300 µL HBSA. After the addition of HBSA (200 µL), 25 µL/well of HBSA (control) or the respective compounds in the described concentration range were added (n = 3). Subsequently, 25 µL/well of [^{125}I]I-TOC (1 nM in HBSA) was added and incubated at RT and protected from light for 1 h. The supernatant solutions were collected, and each cell lawn was washed with ice-cold PBS (300 µL). The wash fractions were combined with each respective supernatant. The cells were lysed by incubating in 300 µL/well of 1 M NaOH at RT for 20 min. The cell lysates were collected, and each well was washed with 1 M NaOH (300 µL). The wash fractions were combined with each respective cell lysate. In a 2480 WIZARD2 γ-counter (PerkinElmer Inc., Waltham, USA), the activities of both the supernatant and lysate were measured. The percentage of [^{125}I]I-TOC activity in the lysate, relative to the total activity in the supernatant and lysate, was plotted against the log of the compound concentration to determine the IC_{50} for Lu-DOTA-TATE and TATE-Ru(II)MT. For the calculation of the SSTR2 IC_{50} values, OriginPro 2021 9.8.0.200 (Origin Lab Corp., Northampton, USA) was used.

Preparation of Tissue Sections

Fresh-frozen NCI-H446 xenograft tumour was donated by the Research Group Imaging and Biomarkers in Oncology, TUM. The tumour was part of the referenced study and was harvested from an eight-week-old athymic nude mouse, Crl:NU/NCr-Foxn1nu, Charles River Laboratories, Sulzfeld.[4] Fresh-frozen murine spleen samples were donated by the Biotechnology and Cell Signalling, Drug Discovery and Development Institute, University of Strasbourg/CNRS (eight-week-old C57BL/6 mouse) and the Pre-clinical Research Group, Translational Oncologic Imaging, TUM University Hospital (15-week-old $p53^{fl/fl}$ mouse). The tissues were embedded in HPMC/PVP-binary hydrogel (75:25) and cut into 7 µm thick sections using a Leica CM1950 cryostat (Leica Biosystems, Nussloch, Germany). The tissue sections were thaw-mounted onto Superfrost glass slides (epredia, Dreieich, Germany) and stored vacuum-packed at -80 °C until further use.

SSTR2 Immunohistochemistry Protocol

Immunohistochemical staining was performed on fresh-frozen tissue sections with 7 µm thickness using the BOND RX^{m} automated staining system (Leica Microsystems, Wetzlar, Germany). All reagents were obtained from Leica unless otherwise stated.

Sections were first washed in BOND Dewax solution (AR9222, Leica Biosystems, Deer Park, USA), followed by rehydration through a graded series of ethanol (100%, 96%, and 70%). Antigen retrieval was performed with BOND epitope retrieval solution 1 (AR9961, Leica Biosystems, Deer Park, USA), corresponding to citrate buffer pH 6, by heat-induced epitope retrieval. Endogenous peroxidase was inactivated by incubation with 3% hydrogen peroxide for 5 min. Sections were incubated with 1:100 clone UMB1 (ab134152, abcam, Cambridge, UK) for 15 min at room temperature. Antibody detection was carried out using the BOND polymer refine detection kit without post-primary reagent (DS9800, Leica Biosystems, Deer Park, USA) for 8 min. Visualisation was achieved using DAB as chromogen (10 min). Sections were counterstained with haematoxylin for 10 min, dehydrated through increasing concentrations of ethanol (70%, 96%, and 100%), cleared in xylene, and mounted using Pertex® mounting medium (00801, HistoLab®, Gothenburg, Sweden) and Leica CV5030 robotic coverslipper (Leica Biosystems, Wetzlar, Germany). Stained slides were scanned using the Aperio AT2 slide scanner

(Leica Biosystems, Wetzlar, Germany) and representative images were taken at 40-fold magnification in Aperio ImageScope software version 12.4 (Leica Biosystems, Wetzlar, Germany).

Mass Tag Staining

All solutions were prepared using analytical grade solvents. Sample preparation was adapted from previously published work.[5]

Briefly, tissue-mounted Superfrost glass slides were initially submerged in pre-chilled ethanol (Sigma-Aldrich, Darmstadt, Germany) at −70 °C for 5 min to achieve rapid fixation. Slides were then air-dried in a desiccator for 10 min before undergoing an additional fixation step in 4% formalin solution (Sigma-Aldrich, Darmstadt, Germany) for 5 min to enhance structural preservation. Following formalin fixation, slides were rinsed in PBS (pH 7.4, Sigma-Aldrich, Darmstadt, Germany) for 10 min and subsequently dehydrated through three sequential washes in 100% ethanol (3 min, 1 min, and 2 min). Carnoy's solution (60% ethanol, 30% chloroform (Carl Roth GmbH + Co. KG, Karlsruhe, Germany) and 10% glacial acetic acid (Alfa Aesar GmbH, Darmstadt, Germany)) was then applied for 3 min to clear the samples and remove residual lipids. After clearing, tissues were rehydrated using graded ethanol washes as follows: 2 × 1 min in 100% ethanol; 2 min in 95% ethanol; 3 min in 70% ethanol, and 3 min in 50% ethanol. Tissues were then washed in Tris-buffered saline (TBS, 50 mM, Promega GmbH, Walldorf, Germany) for 6 min. Heat-mediated antigen retrieval was performed in an alkaline ethylenediaminetetraacetic acid buffer (EDTA, pH 8.5, Sigma-Aldrich, Darmstadt, Germany) at 95 °C for 30 min to unmask protein epitopes. Tissues were then gradually cooled to room temperature through sequential liquid exchanges in TBS, followed by a final 10 min incubation in fresh TBS. The tissue regions were delimited using a hydrophobic (PAP) barrier pen (Sigma-Aldrich, Darmstadt, Germany). To reduce nonspecific antibody binding, tissues were incubated in a blocking solution composed of 2% (v/v) normal rabbit serum (Jackson ImmunoResearch Europe Ltd, Cambridge House, UK), 2% (v/v) normal mouse serum (Jackson ImmunoResearch Europe Ltd, Cambridge House, UK), and 5% (w/v) bovine serum albumin (BSA, Sigma-Aldrich, Darmstadt, Germany), and 0.05% (w/v) octyl β-glucopyranoside detergent (OBG, Sigma-Aldrich, Darmstadt, Germany) in TBS 1 h. After removal of the tissue-blocking solution, tissue regions enclosed by a hydrophobic barrier were covered with the respective staining solution (100 µL/cm^2) and protected from light in a humidified chamber at 4°C throughout the incubation period:

- MALDI-MSI: incubated with a carefully mixed solution of 10 µg/mL TATE-Ru(II)MT and 12 µg/mL Miralys^{TM} COL1A1 (AmberGen Inc., Billerica, USA) in tissue-blocking buffer overnight
- DESI-MSI: incubated with 100 µg/mL TATE-Ru(II)MT in tissue-blocking buffer for 1 h

After the incubation, the staining solution was removed, and tissues were washed by submerging them in washing solutions in a capped polypropylene Coplin staining jar, with the tissue slide positioned face-up horizontally and gently agitated. Sections were washed in TBS for 3 x 5 min and in an aqueous solution of ammonium bicarbonate (50 mM, Sigma-Aldrich, Darmstadt, Germany) for 10 s and 3 x 3 min. Tissues were then dried for 90 min in a vacuum desiccation chamber, protected from light. The MTs were photocleaved by illuminating the dried tissues:

- MALDI-MSI: in the AmberGen Light Box (Ambergen Inc., Billerica, USA) for 2 x 10 min (MALDI-MSI)

- DESI-MSI: at 420 nm with a total radiant power of 2.9 W (93.75 mW/cm^2) for 30 min using a Lumidox® II LED controller and a 96-position single wavelength LED array (analytical, Flanders, USA).

Matrix-Assisted Laser Desorption/Ionisation Mass Spectrometry Imaging

For MALDI-MSI, the slide was coated with 2,5-dihydroxybenzoic acid (DHB, Sigma-Aldrich, Darmstadt, Germany) as the matrix. Matrix spraying was performed using a HTX TM-Sprayer™ (HTX Technologies, LLC, Chapel Hill, USA) with a solution of 15 mg/mL DHB in 70% ethanol and 30% chloroform containing 0.1% trifluoroacetic acid under the following conditions: 70 °C spray temperature, 0.12 mL/min flow rate, 1200 mm/min nozzle velocity, five passes, 2 mm track spacing, 40 mm nozzle height, heal-to-heal pattern, 10 psi N_2 pressure, 2 L/min gas flow, and 10 s drying time between passes. Matrix recrystallisation was performed by attaching the slide to the lid of a Petri dish using magnets and positioning it cut side down toward a filter paper soaked with 1 mL of 5% isopropanol (Sigma-Aldrich, Darmstadt, Germany) placed at the bottom of the Petri dish. The assembly was then heated in an oven at 55 °C for 90 s.

For quality control, a non-tissue region was included in each measurement as a reference. Mass calibration was performed based on the cluster ions of red phosphorus. MALDI-TOF-MSI data acquisition was performed using a timsTOF flex (Bruker Daltonics GmbH, Bremen, Germany) in reflector-positive mode over the *m/z* 500–1500 mass range. 500 shots at 10 kHz were acquired for each measurement, with a laser intensity set to 85%, yielding a lateral resolution of 20 × 20 µm. MSI data were acquired using flexImaging (v. 7.6, build 6) and timsControl (v. 7.0) software (Bruker Daltonics GmbH, Bremen, Germany). The acquired data were loaded into SCiLS Pro (Version 2026a Pro, Bruker Daltonics GmbH, Bremen, Germany) without applying any baseline removal. Feature selection was performed using T-ReX² (QTOF) and an intensity threshold of 0.5%. The data were normalised to the total ion current (TIC) and exported to the .imzML format. LipostarMSI 2.1.0b5 (Molecular Horizon srl., Bettona, Italy)[6] was used for data processing.

Desorption Electrospray Mass Spectrometry Imaging

DESI-MSI was performed on a Q Exactive™ Plus Hybrid Quadrupole-Orbitrap™ mass spectrometer (Thermo Fisher Scientific, Bremen, Germany). Images were recorded in positive ion mode over the *m/z* 80–900 mass range and a lateral resolution of 75 µm. Other settings were: 70,000 mass resolution at *m/z* 200, 250 ms injection time, 320 °C capillary temperature, and S-lens RF setting of 100. For the DESI-sprayer, the following settings were chosen: 1.5 mm sprayer-to-sample distance, 6 mm sprayer-to-inlet distance, 75° spray angle, 10° collection angle, 95% aq. methanol as spray solvent, 1.25 µL/min solvent flow rate, 4.5 kV spray voltage and 7 bar nebulising gas pressure (N_2 purity N5.0). Individual line scans were converted from the raw format to the imzML format using ProteoWizard MSConvert 3.0.23146-4ef0614,[7] and imzMLConverter.[8] MSiReader 0.06 (W.M. Keck Fourier Transform Mass Spectrometry Laboratory, NCSU, USA) was used for data processing.[9]

Figures

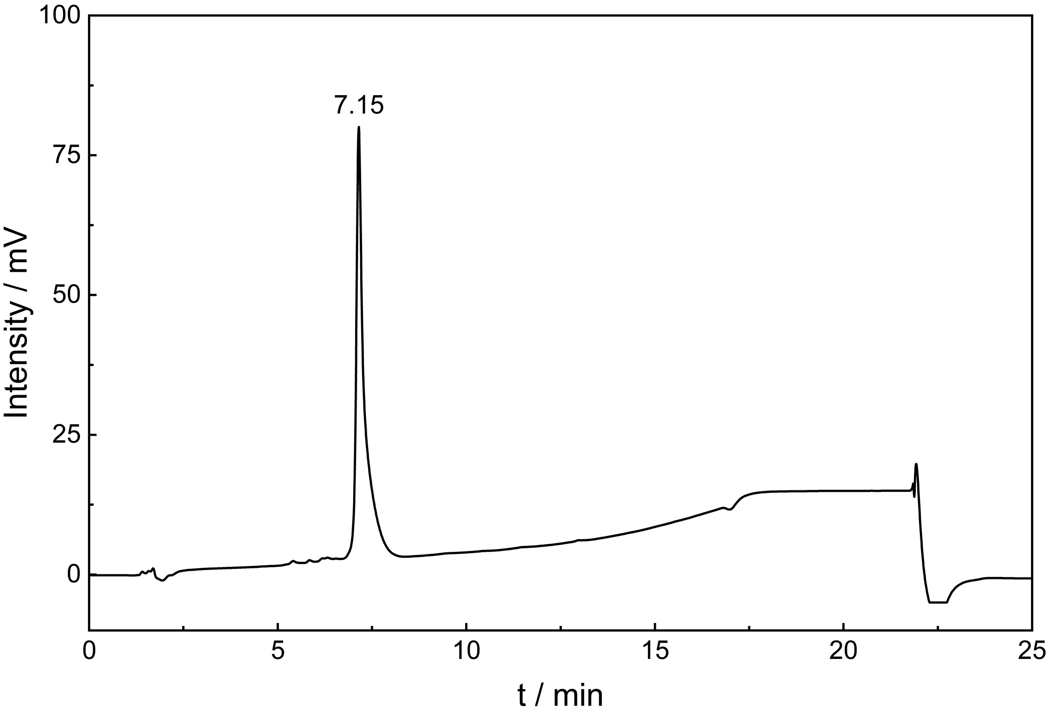

Figure S1. Analytical RP-HPLC chromatogram of **TATE-D-biotin** (10-90% eluent B, 15 min, λ = 220 nm, t_R = 7.15 min).

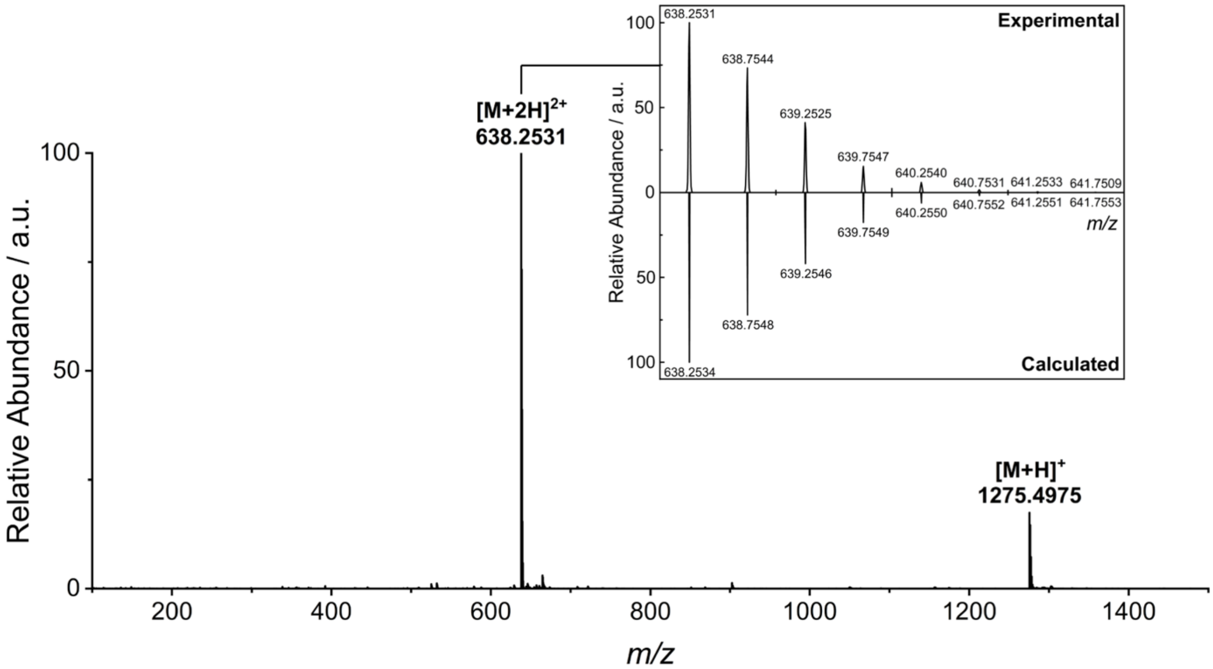

Figure S2. DESI-HRMS spectrum of **TATE-D-biotin**.

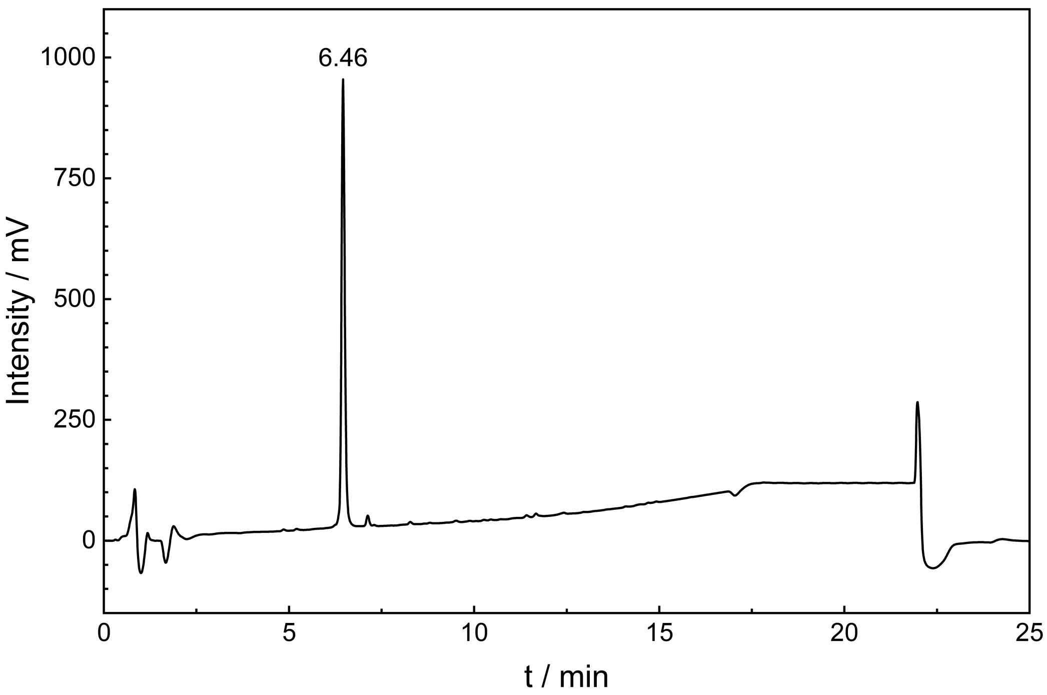

Figure S3. Analytical RP-HPLC chromatogram of **TATE-Ru(II)MT** (10-90% eluent B, 15 min, λ = 220 nm, t_R = 6.46 min).

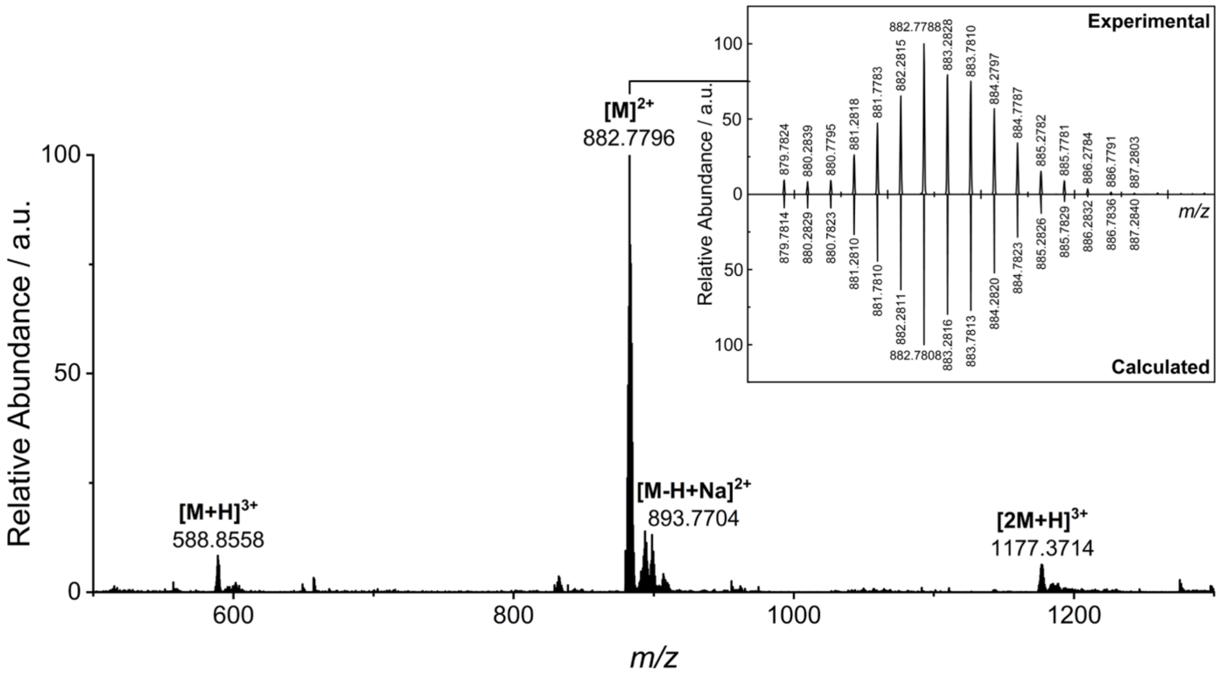

Figure S4. DESI-HRMS spectrum of **TATE-Ru(II)MT**.

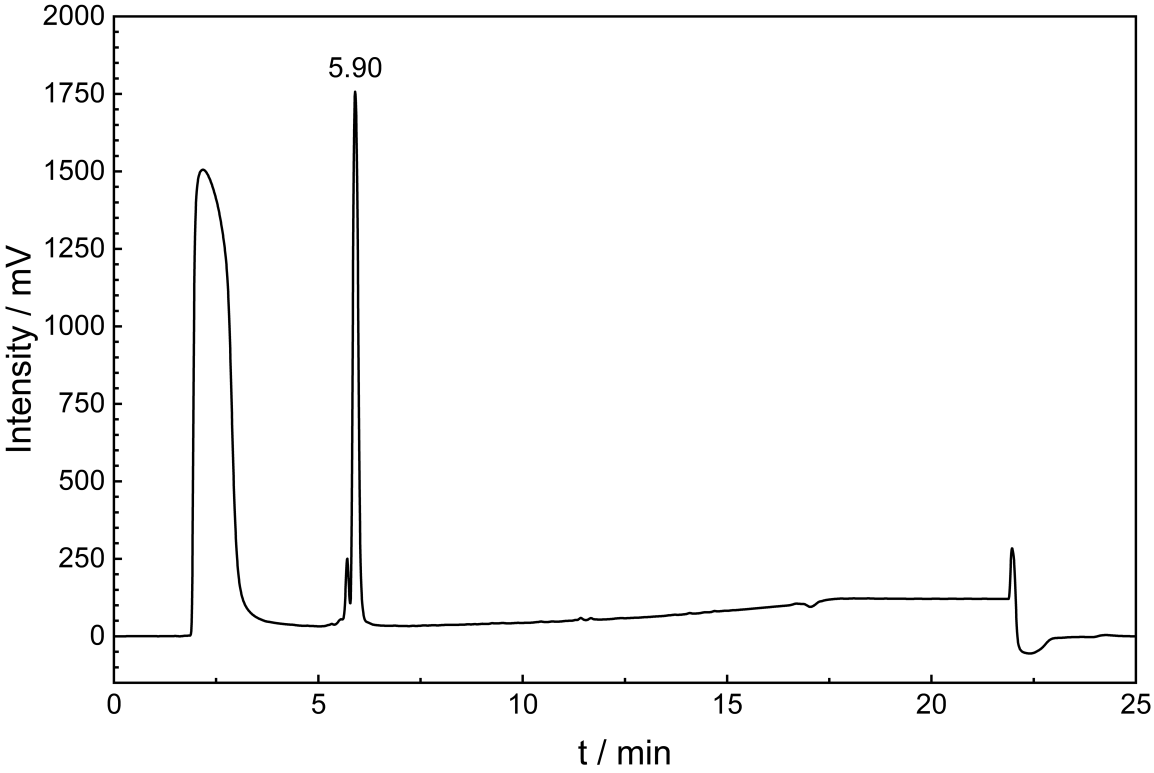

Figure S5. Analytical RP-HPLC chromatogram of **Lu-DOTA-TATE** (10-90% eluent B, 15 min, λ = 220 nm, t_R = 5.90 min).

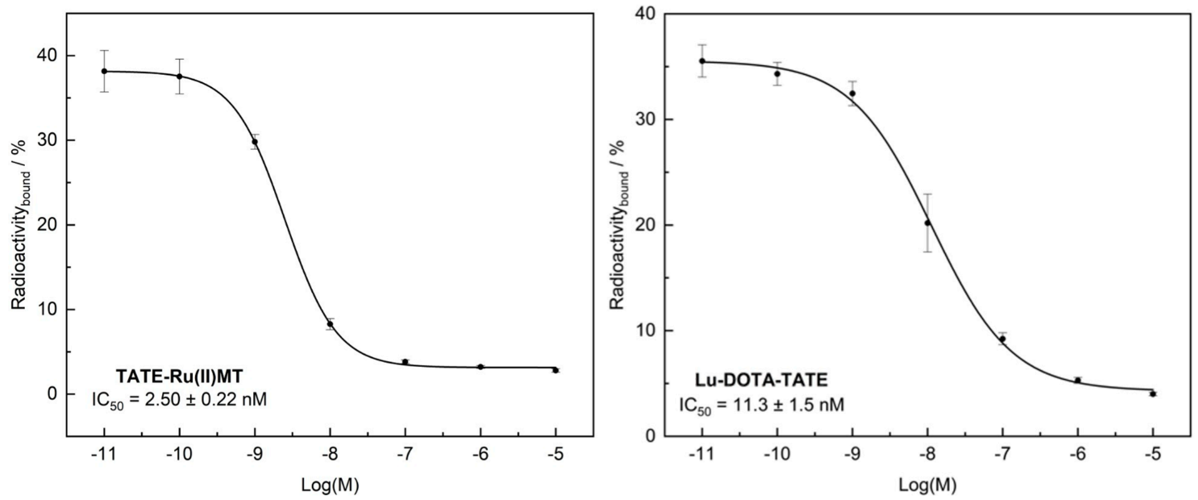

Figure S6. Dose-response curves for determining the binding to SSTR2 (IC_{50} values) of **TATE-Ru(II)MT** and Lu-DOTA-TATE.

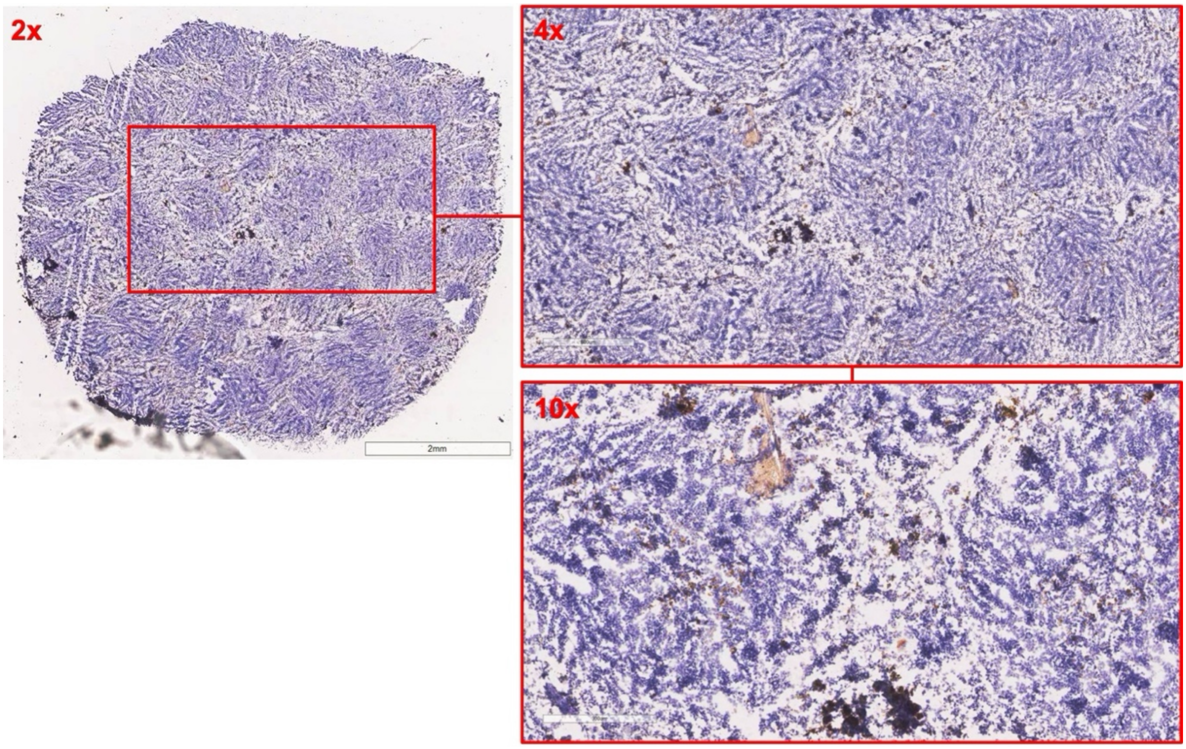

Figure S7. SSTR2 expression in murine spleen (15-week-old p53$^{fl/fl}$ mouse) cross-section assessed by IHC.

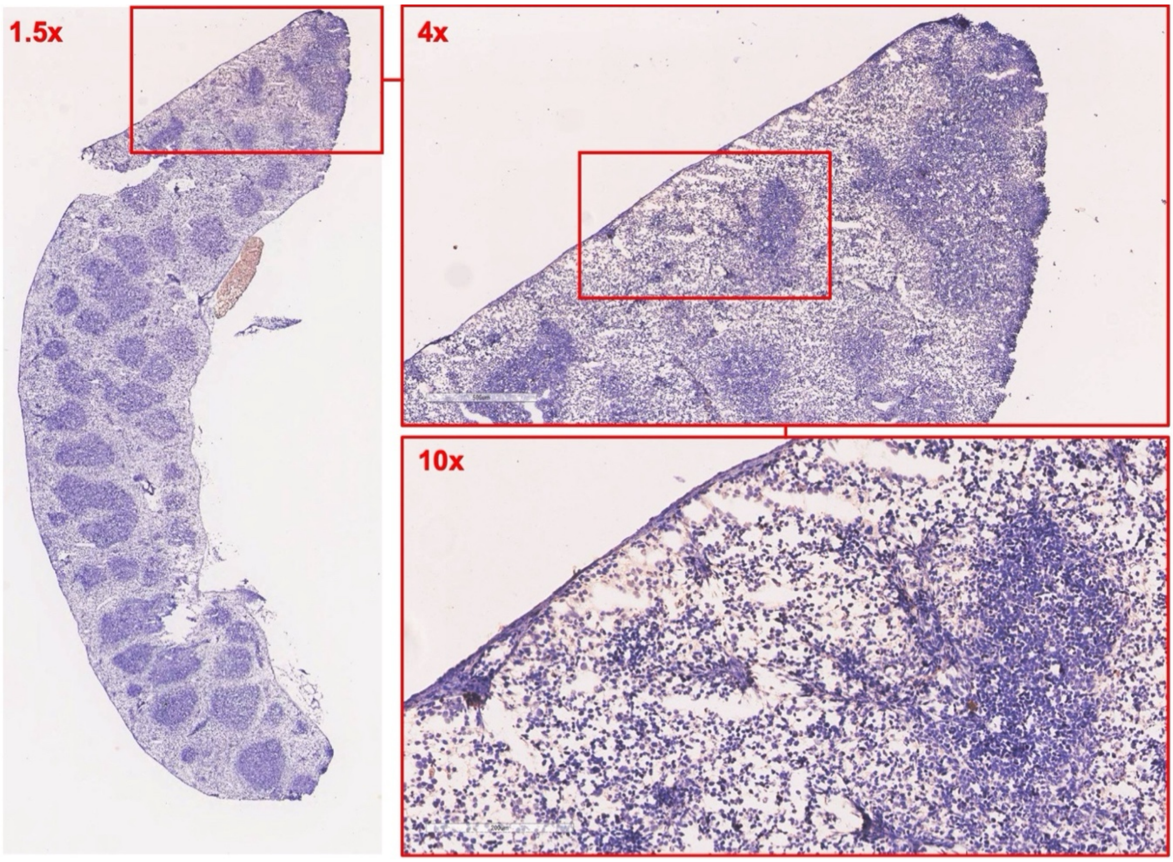

Figure S8. SSTR2 expression in murine spleen (eight-week-old C57BL/6 mouse) longitudinal section assessed by IHC.

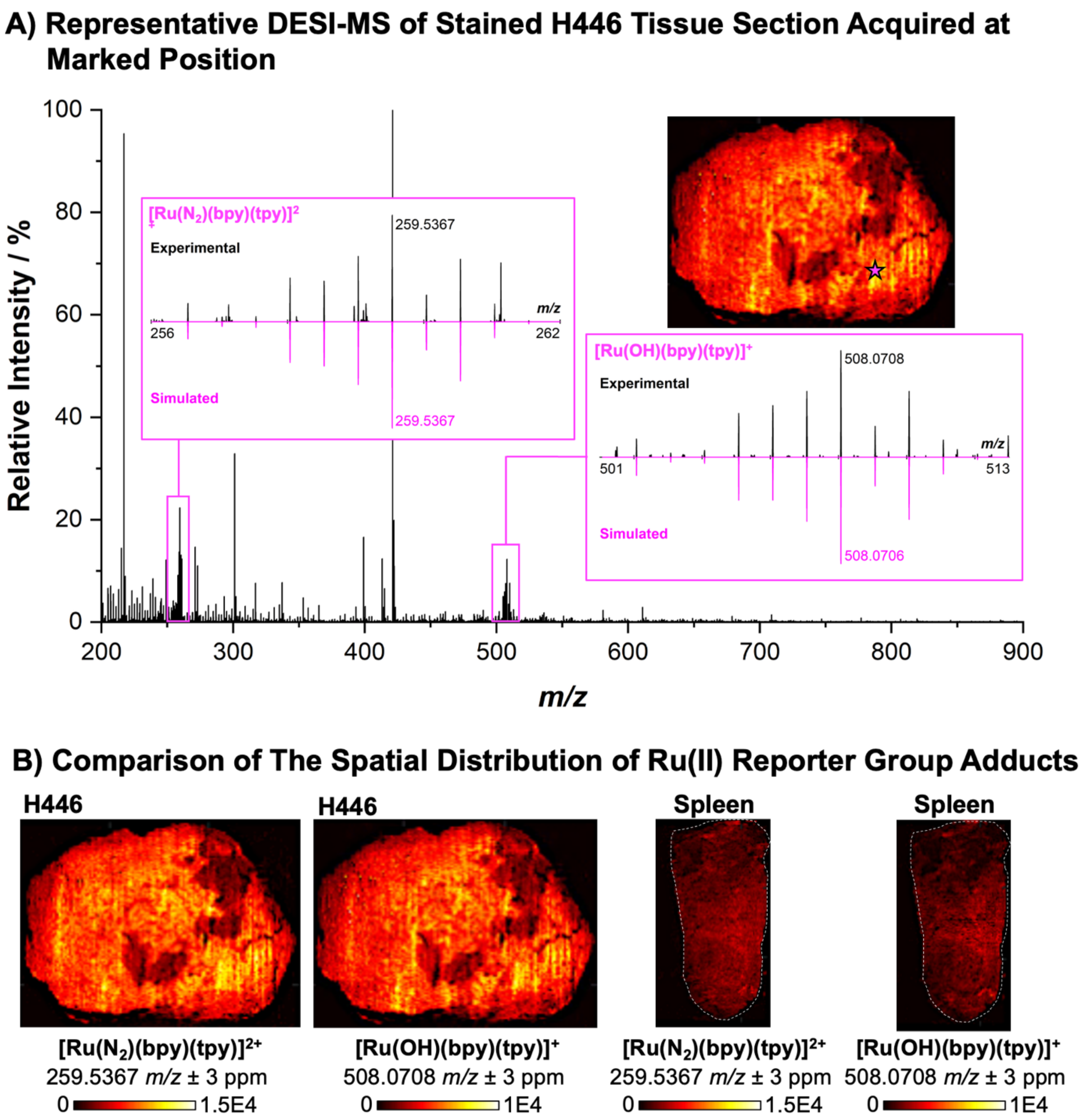

Figure S9. A) Representative DESI-MS of stained H446 tissue section acquired at the marked spot. DESI mass spectrum recorded at the marked spot (pink star) of the H446 xenograft tumour shows the isotope pattern distribution of the two Ru(II) reporter group adducts $[Ru(N_2)(bpy)(tpy)]^{2+}$ (*m/z* 259.5367 ± 3 ppm) and $[Ru(OH)(bpy)(tpy)]^{+}$ (*m/z* 508.0708 ± 3 ppm) in comparison to the simulated isotope patterns. **B) Comparison of the spatial distribution of Ru(II) reporter group adducts.** Similar spatial distribution was observed for $[Ru(N_2)(bpy)(tpy)]^{2+}$ and $[Ru(OH)(bpy)(tpy)]^{+}$ in H466 xenograft tumour and murine spleen sections.

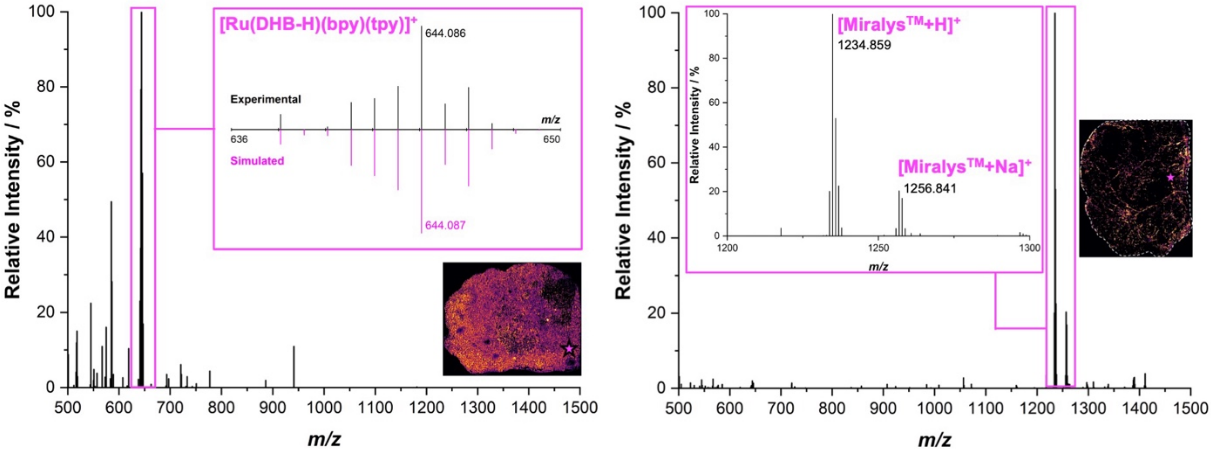

Figure S10. Representative MALDI mass spectra recorded at the marked spots (pink star) of stained H446 xenograft tissue section show the isotope pattern distribution of Ru(II) reporter group adduct $[Ru(DHB\text{-}H)(bpy)(tpy)]^+$ in comparison to the simulated isotope pattern and Miralys^{TM} MT adducts $[Miralys^{TM}+H]^+$ and $[Miralys^{TM}+Na]^+$.

MSI Data

MALDI- and DESI-MSI data files have been provided on Zenodo:

https://doi.org/10.5281/zenodo.18419727

References

1. S. Deiser, S. Fenzl, V. König, M. Drexler, L. M. Smith, M. E. George, R. Beck, T. H. Witney, S. Inoue and A. Casini, (SiFA)SeFe: A Hydrophilic Silicon-Based Fluoride Acceptor Enabling Versatile Peptidic Radiohybrid Tracers, *Journal of Medicinal Chemistry* **2024**, 67, 14077-14094.
2. N. Kaveevivitchai, R. Zong, H.-W. Tseng, R. Chitta and R. P. Thummel, Further Observations on Water Oxidation Catalyzed by Mononuclear Ru(II) Complexes, *Inorganic Chemistry* **2012**, 51, 2930-2939.
3. M. Loos, C. Gerber, F. Corona, J. Hollender and H. Singer, Accelerated Isotope Fine Structure Calculation Using Pruned Transition Trees, *Analytical Chemistry* **2015**, 87, 5738-5744.
4. H. Rauch, C. Kitzberger, K. Janghu, P. Hawarihewa, N. T. Nguyen, Y. Min, S. Ballke, K. Steiger, W. A. Weber and S. Kossatz, Combining [177Lu]Lu-DOTA-TOC PRRT with PARP inhibitors to enhance treatment efficacy in small cell lung cancer, *European Journal of Nuclear Medicine and Molecular Imaging* **2024**, 51, 4099-4110.
5. G. Yagnik, Z. Liu, K. J. Rothschild and M. J. Lim, Highly Multiplexed Immunohistochemical MALDI-MS Imaging of Biomarkers in Tissues, *Journal of the American Society for Mass Spectrometry* **2021**, 32, 977-988.
6. S. Tortorella, P. Tiberi, A. P. Bowman, B. S. R. Claes, K. Ščupáková, R. M. A. Heeren, S. R. Ellis and G. Cruciani, LipostarMSI: Comprehensive, Vendor-Neutral Software for Visualization, Data Analysis, and Automated Molecular Identification in Mass Spectrometry Imaging, *Journal of the American Society for Mass Spectrometry* **2020**, 31, 155-163.
7. R. Adusumilli and P. Mallick, in *Proteomics: Methods and Protocols*, eds. L. Comai, J. E. Katz and P. Mallick, Springer New York, New York, NY, 2017, DOI: 10.1007/978-1-4939-6747-6_23, pp. 339-368.
8. A. M. Race, I. B. Styles and J. Bunch, Inclusive sharing of mass spectrometry imaging data requires a converter for all, *Journal of Proteomics* **2012**, 75, 5111-5112.
9. G. Robichaud, K. P. Garrard, J. A. Barry and D. C. Muddiman, MSiReader: An Open-Source Interface to View and Analyze High Resolving Power MS Imaging Files on Matlab Platform, *Journal of The American Society for Mass Spectrometry* **2013**, 24, 718-721.
